# The Foraging Ecology of the Endangered Cape Verde Shearwater, a Sentinel Species for Marine Conservation off West Africa

**DOI:** 10.1371/journal.pone.0139390

**Published:** 2015-10-05

**Authors:** Vitor H. Paiva, Pedro Geraldes, Isabel Rodrigues, Tommy Melo, José Melo, Jaime A. Ramos

**Affiliations:** 1 MARE - Marine and Environmental Sciences Centre, Department of Life Sciences, University of Coimbra, Coimbra, Portugal; 2 SPEA - Sociedade Portuguesa para o Estudo das Aves, Lisboa, Portugal; 3 Biosfera I, Mindelo, São Vicente, Cabo Verde; Institut Pluridisciplinaire Hubert Curien, FRANCE

## Abstract

Large Marine Ecosystems such as the Canary Current system off West Africa sustains high abundance of small pelagic prey, which attracts marine predators. Seabirds are top predators often used as biodiversity surrogates and sentinel species of the marine ecosystem health, thus frequently informing marine conservation planning. This study presents the first data on the spatial (GPS-loggers) and trophic (stable isotope analysis) ecology of a tropical seabird—the endangered Cape Verde shearwater *Calonectris edwardsii*–during both the incubation and the chick-rearing periods of two consecutive years. This information was related with marine environmental predictors (species distribution models), existent areas of conservation concern for seabirds (i.e. marine Important Bird Areas; marine IBAs) and threats to the marine environment in the West African areas heavily used by the shearwaters. There was an apparent inter-annual consistency on the spatial, foraging and trophic ecology of Cape Verde shearwater, but a strong alteration on the foraging strategies of adult breeders among breeding phases (i.e. from incubation to chick-rearing). During incubation, birds mostly targeted a discrete region off West Africa, known by its enhanced productivity profile and thus also highly exploited by international industrial fishery fleets. When chick-rearing, adults exploited the comparatively less productive tropical environment within the islands of Cape Verde, at relatively close distance from their breeding colony. The species enlarged its trophic niche and increased the trophic level of their prey from incubation to chick-rearing, likely to provision their chicks with a more diversified and better quality diet. There was a high overlap between the Cape Verde shearwaters foraging areas with those of European shearwater species that overwinter in this area and known areas of megafauna bycatch off West Africa, but very little overlap with existing Marine Important Bird Areas. Further investigation on the potential nefarious effects of fisheries on seabird communities exploiting the Canary Current system off West Africa is needed. Such negative effects could be alleviated or even dissipated if the ‘fisheries-conservation hotspots’ identified for the region, would be legislated as Marine Protected Areas.

## Introduction

Tropical marine ecosystems are generally oligotrophic (i.e. nutrient-poor waters) environments when compared to higher latitude temperate and eutrophic regions [1]. As a consequence, prey fish are usually patchily distributed and low in abundance. To cope with this, some seabird species have evolved a dual-foraging strategy, alternating from short foraging trips exploiting the less productive colony surroundings, to long excursions searching for prey at distant, more productive, regions [2]. Profitable environments are commonly located on neritic and coastal regions, some designated as Large Marine Ecosystems (LMEs), such as the Canary Current (CC) system off West Africa [1]. Here, the strong, constant and nutrient-rich upwelling phenomena (i.e. sea surface with low temperature and high chlorophyll *a* concentration), congregates and sustains high abundance of small pelagic prey, which attracts not only aerial (e.g. [3]) but also aquatic (e.g. [4]) marine predators. The region is also highly targeted by industrial fisheries, and has been recently identified as one of the World’s ‘fisheries-conservation hotspot’, i.e. a region of increasing exploitation rates, high marine biodiversity, and poor management capacity [4]. Indeed, the huge level of Illegal Unreported and Unregulated (IUU) catches and fishing quotas established beyond scientific advice, might be jeopardizing the subsistence of this very profitable LME in the near future [5–7].

The use of miniaturized tracking devices (such as global positioning system—GPS—devices; GPS-loggers) in combination with stable isotope analysis (SIA), has become a powerful tool to study in an holistic manner the spatial and trophic ecology of marine apex predators, such as seabirds. Highly precise positioning data provided by GPS-loggers, allows a good interpretation of the spatio-temporal scale that marine predators use to encounter their prey (e.g. [2]), i.e. an understanding of how animals perceive the hierarchical structure of the marine environment [8], by increasing residence time within productive patches (Areas of Restricted Search—ARS; [9]). On the other hand, SIA is based on the assumption that the isotopic signature of predators is directly influenced by what they consume [10]. Hence, the stable carbon signature of consumers is similar to that of their diets, thus making it a useful tool to identify foraging regions, while the nitrogen signature reflects the predators’ trophic position, with a stepwise increase at each trophic level [10]. Furthermore, because animal tissues are synthetized in a predictable manner and have different turnover rates, we can investigate the consumers’ dietary choices from the previous weeks (whole blood) to months (new growing feathers after moult) [11].

Seabirds are frequently used as biodiversity surrogates, i.e. their foraging distribution likely represent critical ‘hotspots’ of productivity, which often overlap with fisheries, leading to potential competition for marine resources [12]. For seabirds, the impacts of this spatio-temporal competition for resources usually comprise a decrease in resources availability and alteration of the trophic balance in the environment (indirect effects; e.g. [13]) and a possible increase in accidental mortality of birds by-caught in fishing gears (direct effect; e.g. [14]). The identification of a ‘fisheries-conservation hotspot’ off West Africa, where marine megafauna might be at risk of survival, already grasped the attention of conservationists and researchers [15,16]. This is a relevant motive of concern for marine wildlife conservation in general, and particularly for species of conservation concern, such as the Cape Verde shearwater *Calonectris edwardsii* (Near Threatened, [17]). This endemic species from the Cape Verde archipelago likely forages off West Africa while breeding [18]. Direct observations of feeding events suggest that they may rely to some extent on easy meals supplied by fisheries subsidies [18], which are typically composed by low lipid content prey. At first sight, these might seem a suitable alternative to ‘natural’ lipid-rich preys, but in the mid- to long-term are negative to individual fitness [19] and is reported to have an immediate negative impact on the growth of Cape gannet *Morus capensis* chicks [20]. This is in line with the effects described by the ‘junk-food hypothesis’ for seabirds feeding on fishery waste [19]. Previous dietary studies indicate Cape Verde shearwaters feed on the most abundant commercial fish species, such as sardinella *Sardinella* sp, bigeye scad *Selar crumenophthalmus* or scad *Decapterus sp* species, and non-commercial prey, like keelted needlefish *Platybelone argalus lovii* or squid *Loligo sp* [21,22]. Yet, diet composition should be further investigated in the near future, gathering more robust sample sizes and thus corroborating (or not) the consumption of fishery discards by the species (e.g. demersal low lipid content prey, such as Senegalese hake *Merluccius senegalensis*).

The total population of Cape Verde shearwaters was estimated at ~10 000 pairs [17], with ~6500 pairs breeding at Raso Islet (16°36’40.63”N, 24°35’15.81”W), Cabo Verde archipelago (Biosfera I, unpublished data). The species lays one single egg in early June with no clutch replacement. The incubation period lasts for approximately 2 months and is shared between males and females [23]. Both parents feed the chick for about 2 months, and the regularity of chick provisioning decreases as the season progresses [23]. Anecdotal information suggest the population has been declining, owing to uncontrolled levels of chicks harvesting. In 2006, ~6000 chicks were killed just at Raso Islet, which was presumably and historically the typical amount of chicks to harvest yearly (Biosfera I, unpublished data) and represented ~92% breeding failure. Only since 2008, Biosfera I has guaranteed through surveillance that virtually no chick is killed at that islet. Adding to the former threats at their breeding grounds and surrounding at-sea regions, Cape Verde shearwaters like other long-distance migratory seabirds, face threats over large geographical ranges, particularly along their main migratory routes [24] to achieve their non-breeding region off south Brazil [25]. Threats such as being by-caught on fishing gears [26] or suffering contamination from marine pollutants [27]. Despite former contributions to the study of the species migratory patterns [24], non-breeding foraging [28] and trophic [29] ecology, there is virtually no information on the species’ spatial and trophic ecology during the breeding phase. Given its marine top predator status, relative abundance, size (i.e. enabling to carry non-expensive GPS devices), easy access to breeding colonies, low fecundity and overall high sensitivity to Human-induced alterations to the marine ecosystem off West Africa, Cape Verde shearwater is an ideal sentinel species of the health of such ecosystem.

This work addresses, for the first time, the spatial ecology (GPS trackers) and trophic niches (isotopic signatures) of Cape Verde shearwaters during the incubation and chick-rearing periods of two consecutive years. Our purpose was to examine the at-sea distribution, behaviour and trophic ecology of this near threatened species [17], relate this with marine environmental predictors (e.g. Sea Surface Temperature; SST), existent areas of conservation concern for seabirds (i.e. marine IBAs), the distribution of other seabird species and threats to the marine environment in the west African areas heavily used by the shearwaters. We specifically aimed to answer a two-fold group questions: (1) at-sea distribution and habitat use by Cape Verde shearwaters: (1a) Do they use the same areas during the incubation and chick-rearing periods and between years? (1b) Which environmental predictors best characterize foraging areas? (1c) Do birds alter their isotopic niche, from incubation to chick-rearing and between study years? We expect incubating adults to target high productive foraging patches likely at distance from their colony, within the CC system [2] and feeding (until some extent) on food subsidies from fishery discards. Thus their trophic niches should depict this behaviour (i.e. high δ^15^N values shaped by the consumption of demersal species) in accordance with the ‘junk-food hypothesis’. This hypothesis attributes declines in the productivity of seabirds to a diet of low nutritional quality, such as that based on discarded fish. Fishery discards are mostly composed by demersal species that tend to have a low lipid content, when compared to ‘natural’ lipid-rich prey species (e.g. keelted needlefish). During chick-rearing Cape Verde shearwaters should be more constrained to be central-place foragers, thus having to find productive patches at short distance from their breeding colony [30]. According to [31], parents should select high quality food (i.e. high trophic level prey) to bring to their growing chick [32], and thus we expect an increase of trophic level from incubation to chick-rearing. (2) Relationships between the at-sea distribution of Cape Verde shearwaters and marine conservation off West Africa: (2a) How the distribution of the species compares with that of other ‘GPS-like’ tracking information available on the literature (namely the distribution of northern gannets *Morus bassanus* [16] and Scopoli’s shearwaters *Calonectris diomedea* [33])? (2b) Do areas heavily used by the birds coincide with previously identified Marine Important Bird Areas for other seabird species? (2c) What are the most important threats for the conservation of marine biodiversity within the southern branch of the CC system, around Cape Verde Islands and off West Africa, where seabirds might be at threat from marine plundering [16]?

## Methods

### Ethics statement

The deployment of GPS-loggers (see details below) did not take more than 10 minutes and on no occasion did it interfere with reproduction or have visible deleterious effects on study animals. All work on Raso Islet was approved and certified by annual permits (P2013, P2014) issue by ‘Direcção Geral do Ambiente de Cabo Verde’ (DGACV; environment governmental authority of Cape Verde). No animal ethics committee approval was required by DGACV. all sampling procedures and/or experimental manipulations were reviewed and specifically approved as part of obtaining the field permit.

### Birds instrumentation and tracking data

The tracking study was performed on Raso Islet (16°36’40.63”N, 24°35’15.81”W) located at ~16km from S. Nicolau Island, Cape Verde archipelago, during mid-June (incubation data) and mid-September (chick-rearing data; when chicks were ~six weeks old) of 2013 and 2014. GPS tracking devices CatTraq Travel Loggers (Perthold Engineering LLC) were employed as GPS-loggers. This device (44.5 * 28.5 * 13mm) weighs 13g and contains a SiRF StarIII chipset, a patch antenna and a 180mAh Lithium-ion battery. Devices were sealed with a thermo-retractile rubber sleeve for waterproofing. Loggers weight represented between 1.8% and 2.8% (median = 2.5%) of the birds weight. Devices were set to record data each 5 minutes, with loggers’ batteries draining out in about 15 days. Birds were captured during the night at their nest sites, weighed and individually identified by their ring numbers. GPS loggers were then attached using TESA^®^ tape to the contour feathers along and in between both scapulas. Total handling time did not exceed 10 minutes and birds were released immediately after. At logger retrieval, a blood sample of about 150μl was collected from the tarsal vein of each individual, for evaluation of its trophic choices during the tracking period, through stable isotope analysis (SIA). Sex of the processed individuals was also annotated based on their distinguishable vocalizations (i.e. higher pitched vocalizations of males when compared to females).

### Area of Restricted Search (ARS) zones

Fauchald & Tveraa (2003) developed a technique, named First Passage Time (FPT) to assess the spatial scale that animals use to encounter their prey. FPT is, by definition, the time required for an animal to pass through a circle with a given radius *r*. By moving this circle along the path of the animal, we will obtain a scale-dependent measure of search effort and therefore the behavioural response of an individual in the environment. Because top marine predators usually forage in a patchy and hierarchical environment [9], increases in the turning rate and/or decreases in speed of its foraging path should be related to the so-called Area Restricted Search behaviour (ARS). ARS will then appear as an individual reaction to changes in the resources availability and distribution, by increasing the residence time in the productive patch [34].

Zones of ARS were estimated applying FPT analysis, following [34] and using software R 3.0 (R Development Core Team 2011). Usually, ‘in water’ positions result in very small-scale ARS zones (<100 m diameter), which considerably increase the variance in FPT and can camouflage larger-scale ARS zone [35]. To address this problem, we removed bouts on the water and interpolated locations to obtain a distance interval of 0.1 km for FPT analysis [36]. We considered positions with speed < 3 km h^−1^ as resting or preening behaviours on the water or inland, after inspection of the frequency distribution of speeds. Following the recommendations of [36], FPT analysis was performed in two steps: 1) to detect large-scale ARS we run the analysis on the whole path, estimating the FPT every 1 km for a radius *r* from 1 to 50 km; 2) to detect small spatial scale events we run again FPT analysis every 0.1 km for an *r* varying between 0.1 and 10 km. The plot representing variance in log (FPT) as a function of *r* allowed us to identify the ARS scales by peaks in the variance. In this calculation, FPT was log transformed to make the variance independent of the magnitude of the mean FPT [34]. It is also possible to locate where the bird entered an ARS zone and the time spent on that area by plotting FPT values where a peak of variance occurred as a function of time since departure from the colony. ARS locations were also used to feed the *habitat use* and *habitat suitability model* analysis methods.

### Habitat use

GPS locations of each bird where ARS behaviour was detected (ARS zones) were examined under the *adehabitatHR* R package [37] generating Kernel Utilization Distribution (Kernel UD) estimates. The most appropriate smoothing parameter (*h*) was chosen via least squares cross-validation for the unsmoothed GPS data, and then applied as standard for the other datasets and grid size was set at 0.12° (to match the coarsest grid of the environmental predictors). We considered the 50% and 95% kernel UD contours to represent the core foraging areas (FR) and the home range (HR), respectively. The overlap between kernel FRs (50% kernel UDs) of different (1) years and (2) breeding stages were computed to study the spatial segregation within and among groups with the *kerneloverlap* function and *VI* method of the *adehabitatHR* library [37].

### Habitat suitability models

#### Environmental predictors

To characterize the oceanographic conditions in areas used by the tracked individuals we extracted: (1) Bathymetry (BAT, blended ETOPO1 product, 0.01° spatial resolution, m), (2) Sea Surface Temperature (SST, Aqua MODIS NPP, 0.04°, °C), (3) sea surface Chlorophyll *a* concentration (CHL, Aqua MODIS NPP, 0.04°, mgm^−3^), gradients in these 3 variables–(4) BATG, (5) SSTG and (6) CHLG, respectively—and (7) wind speed (WSPD, QuickSCAT, 0.12°, ms^−1^). Variable 1 was downloaded from http://ngdc.noaa.gov/mgg/global/global.html, variables 2 and 3 were extracted from http://oceancolor.gsfc.nasa.gov, while variable 7 was downloaded from http://winds.jpl.nasa.gov. Monthly averages were used for the dynamic variables (variables 2, 3 and 5–7). Gradients were determined by estimating rates of change by moving a window function (3 x 3 grid cells; function = [(max. value − min. value) × 100] / (max. value)). Fronts, as zones of strong CHL variations, will appear more clearly when using CHLG than using CHL values alone. Gradient in depth (BATG) was used as a proxy of slope. Distance to colony (DCOL) was computed as the minimum direct distance to colony. All environmental predictors were gathered to the coarsest grid cell (0.12°).

#### Data processing and exploratory analysis

To minimize the influence of any particular individual on each model, we randomly selected an equal number of ARS locations for each bird during a specific phase (incubation and chick-rearing period) and study year (2013 and 2014), based on a bootstrapping procedure [38,39]. All 8 predictor variables for each breeding stage were inspected under MaxEnt Model Surveyor (MMS; http://phycoweb.net/software/MMS/index.html), which automatically computed the Akaike and Bayesian information criteria (AIC, BIC; [40]) and the test AUC under the various predictor sets and suggested "suitable" predictor sets for our dataset [41], thus avoiding including highly correlated variables on our models.

#### Model evaluation and calibration

Model construction, training and testing was performed with Maximum Entropy (MaxEnt) modelling based on presence-only data ([42]; version 3.3.3 (http://www.cs.princeton.edu/~schapire/maxent/). The MaxEnt method does not require absence data for the species being modelled; instead it uses background environmental data from the entire study area. This method has been shown to perform well in comparison with alternative methods [43] and when modelling habitat use from tracking data (e.g. [39,44,45]). ARS locations were divided into training and test data by setting aside approximately 30% of the ARS locations dataset for spatial evaluation of the models [46]. We ran MaxEnt on the presence-only positions 50 times. We calculated the mean of the 50 MaxEnt predictions to obtain an average prediction and coefficient of variation of predictions [38]. The MaxEnt program was run separately for different years (2013 and 2014) and breeding phases (incubation and chick-rearing), totalizing four habitat models. The settings were logistic output format, resulting in values between 0 and 1 for each grid cell, where higher values indicate more similar climatic conditions, duplicates removed, and 50 replicate runs of random (bootstrap) subsamples with 30 as random test percentage. The results were summarized as the average of the 50 models.

From the MaxEnt main results, the Jackknife chart was used to evaluate the contribution of each environmental layer to the final result, thus providing the explanatory power of each variable when used in isolation. The ROC curve was used to assess the model’s accuracy, as measured by the Area Under the ROC Curve (AUC). The AUC estimates the likelihood that a randomly selected presence point is located in a raster cell with a higher probability value for species occurrence than a randomly generated point [42]. Generated models are generally interpreted as excellent for test AUC > 0.90, good for 0.80 < AUC < 0.90, acceptable for 0.70 < AUC < 0.80, bad for 0.60 < AUC < 0.70 and invalid for 0.50 < AUC < 0.60. All model evaluation statistics and optimal thresholds were calculated using the package *PresenceAbsence* in R [47].

### Trophic ecology

We performed Stable Isotope Analysis (SIA) on whole blood for δ^15^N (^15^N/^14^N) and δ^13^C (^13^C/^12^C) in order to estimate the trophic positioning and the foraging habitat of the tracked birds, respectively. Nitrogen is enriched at each successive trophic level by 2 to 5‰, whereas carbon is enriched (~0.8‰) when foraging in coastal or benthic areas in relation to offshore or pelagic areas [48]. Whole blood (WB) should retain the dietary choices of individuals of the last four weeks prior to sample collection, thus depicting the trophic ecology during the incubation and chick-rearing periods [49]. WB samples were then dried at 60°C for 24 h and then homogenized. The carbon and nitrogen isotopic composition of the samples were determined under a mass spectrometer (Thermo Delta VS). Replicate measurements of internal laboratory standards (acetanilide) indicate precision < 0.2‰ for both δ^13^C and δ^15^N.

### Data analysis

#### At sea-habitat use and trophic ecology of Cape Verde shearwater

Generalized Linear Mixed Models (GLMMs; *lme4* package; [50]) were used in all statistical analysis, including trip identity nested within the individual as a random term to account for pseudo-replication issues. Response variables were visually tested for normality (through Q-Q plots) and homoscedasticity (using Cleveland dotplots) [51] before each statistical test and were log-transformed when needed. After transformation, the data and the error structure approached the normal distribution, and therefore a Gaussian family (link = “identity”) was selected for all models [52]. Because some habitat conditions (e.g. SST) may change between breeding phases and study years, we expect birds to exhibit phase specific movements and strategies that should result in an improved exploitation of marine resources. Selection of the best procedure to apply under the GLMM framework was made following the decision tree for GLMM fitting and inference and advices from [53]. GLMMs tested the effect of sampling year (2013 *vs* 2014) and breeding phase (incubation *vs* chick-rearing) on mean foraging trip characteristics (e.g. trip duration), spatial ecology parameters (e.g. ARS radii) and trophic signatures of Cape Verde shearwaters. Initially, sex was tested as a factor but dropped from all models due to it’s lack of significance (all models: p > 0.18).

The Stable Isotope Bayesian Ellipses in R (SIBER) were used to establish the isotopic niche of both groups among periods [54]. The area of the standard ellipse (SEA_C_, an ellipse that has 95% probability containing a subsequently sampled datum) was adopted to compare isotopic signatures between years (2013 and 2014) and breeding phases (incubation and chick rearing), and their overlap in relation to the total niche width (both groups combined), and a Bayesian estimate of the standard ellipse and its area (SEA_B_) to test whether group 1 is smaller than group 2 (i.e. *p*, the proportion of ellipses in incubation that were lower than in chick rearing; see [54] for more details). All the metrics were calculated using *standard*.*ellipse* and *convexhull* functions from the *SIAR* package (Stable Isotope Analysis in R; [55]). All statistical analyses were performed within the R environment [47]. Data is shown as mean ± 1 SD, unless otherwise stated. Results were considered significant at *P* ≤ 0.05.

#### Foraging distribution of the Cape Verde shearwater and marine conservation

The *kerneloverlap* function (*adehabitatHR* library [37]) was also used to measure the overlap between the FR (50% kernel UD) contours of Cape Verde shearwaters during incubation and chick-rearing of both study years and (1) foraging distribution of other seabird species using the West African area (only precise GPS tracking data), namely juvenile northern gannets (GPS/PTT tags; [16]) and juvenile, immature and adult Scopoli’s shearwaters (GPS/PTT tags; [33]); (2) confirmed, proposed or candidate marine IBAs (http://maps.birdlife.org/marineIBAs/default.html), as broad areas of conservation concern for seabird; (3) identified areas of megafauna bycatch (e.g. turtles, rays, sharks, dolphins, whales; [56]) and foreign licence fishing region [57].

## Results

### Foraging patterns

**Fig 1:**
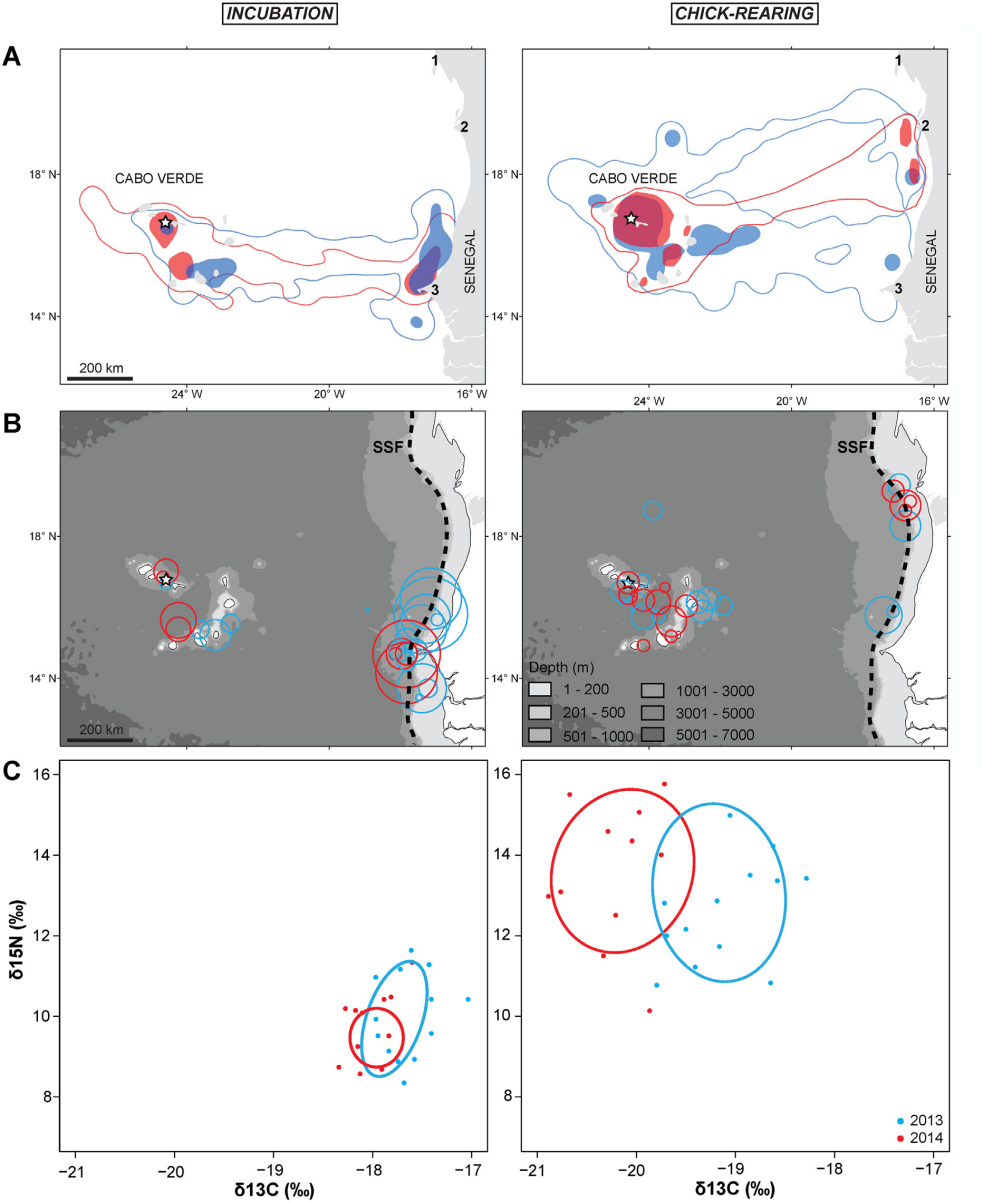
**(A)** Home range (95% kernel UD; lines) and core Foraging areas (50% Kernel UD; filled polygons) of Cape Verde shearwaters *Calonectris edwardsii* from Raso Islet (white star) in 2013 (blue; N = 69 trips from 22 ind.) and 2014 (red; N = 68 trips from 21 ind.). 1 –Cap Blanc; 2 –Southernmost area of the Parc National Du Banc D’Arguin; 3 –Cap-Vert, Dakar, Senegal. **(B)** Areas of Restricted Search zones (ARS; circles) of birds in 2013 (blue) and 2014 (red). Circles represent the ARS zones with maximum First Passage Time (FPT; with size proportionate to the size of ARS zone). SSF—Shelf-Slope Front. **(C)** Isotopic niche area based on stable isotope ratios (δ^13^C and δ^15^N) in whole blood of birds in 2013 (*blue dots*) and 2014 (*red dots*). The Standard ellipses areas (SEAc) are represented by the *solid bold lines* (see Jackson et al. 2011 for more details on these metrics of isotopic niche width).

Cape Verde shearwaters exhibited an overall high inter-annual constancy on their foraging distribution, both during incubation and chick-rearing, whilst there was a noticeable shift in the foraging distribution of individuals between breeding phases (Fig 1A). During incubation, birds mostly target a discrete region off West Africa (in front of Dakar, Senegal), foraging over the shelf and shelf break of the African continent. Such long trips (> 3 days of duration; based on the frequency of occurrence of trip duration) represented 76.2% and 73.6% in relation to 23.8% and 26.4% of short trips (≤ 3 days of duration), performed respectively in 2013 and 2014. When rearing their chick, birds mostly foraged within their colony surroundings, exploiting shallower areas within the Cape Verde Islands, with very few trips towards the African coast, again foraging over the shelf break but further north in the African shelf. During this period, short trips represented 77.1% and 77.5% in relation to 22.9% and 22.5% long excursions, respectively for 2013 and 2014. Overall, off West Africa birds confined their distribution between the Southernmost area of the ‘Parc National Du Banc D’Arguin’ and the ‘Cap-Vert’ in front off Dakar, Senegal, foraging over the Shelf-Slope Front (Fig 1A and 1B).

Foraging trip characteristics varied little between the two study years (F < 2.75, P > 0.10), but were generally significantly different between breeding stages (Tables 1 and 2). During incubation, birds flew more time per day, during more days, covering longer distances and foraging farthest from their colony, when compared to the chick-rearing period. Plus, ARS zones at both meso- and coarse- scales were larger, located furthest from their colony and birds spent more time (higher FPT) inside those areas when compared to the chick-rearing period (Tables 1 and 2).

**Table 1:** Mean foraging trip characteristics, spatial and trophic ecology parameters of Cape Verde shearwaters *Calonectris edwardsii* from Raso Islet (Cape Verde archipelago). FR—Foraging Region; 50% Kernel UD. Values are mean ± SD.

	Incubation		Chick-rearing	
Variables	2013	2014	2013	2014
**Foraging trip characteristics**				
N tracks [N birds]	21 [12]	19 [10]	48 [10]	49 [11]
Trip duration (days)	8.1 ± 3.6	7.6 ± 4.2	1.9 ± 0.9	2.0 ± 0.7
Total distance covered (km)	1943.8 ± 572.5	1899 ± 463.2	596.4 ± 201.1	426.2 ± 146.0
Maximum distance from colony (km)	733.4 ± 183.1	739.7 ± 124.0	187.0 ± 47.4	195 ± 54.4
Time spent flying trip^−1^ day^−1^(h)	6.2 ± 1.5	5.8 ± 1.8	8.7 ± 1.4	9.1 ± 1.8
**Spatial ecology parameters**				
Meso-scale Area of Restricted Search (ARS) Radii (km)	73.3 ± 6.2	70.1 ± 5.4	19.5 ± 4.4	20.4 ± 3.9
Meso-scale max. First Passage Time (hours)	38.4 ± 5.4	29.1 ± 5.8	10.2 ± 2.1	12.8 ± 1.3
Distance of meso-scale ARS zone to colony (km)	710.2 ± 122.4	719.3 ± 108.2	134.2 ± 31.2	125.0 ± 48.6
Coarse-scale Area of Restricted Search (ARS) Radii (km)	13.5 ± 2.4	14.3 ± 3.8	1.9 ± 0.6	2.6 ± 1.9
Coarse-scale max. First Passage Time (hours)	11.8 ± 3.7	12.3 ± 3.3	5.5 ± 2.7	7.3 ± 3.7
Distance of coarse-scale ARS zone to colony (km)	598.1 ± 98.7	609 ± 101.0	58.3 ± 17.7	67.3 ± 21.5
FR overlaps within years and within the same breeding stage (%)	91.0 ± 54.2	87.2 ± 47.2	72.2 ± 39.2	69.6 ± 42.5
FR overlaps among years and within the same breeding stage (%)	85.8 ± 9.1	—	69.9 ± 11.1	—
FR overlaps within breeding stages (%)	84.6 ± 29.9		77.9 ± 27.1	
FR overlaps among breeding stages (%)	18.4 ± 12.5			
**Trophic ecology**				
δ^15^N (‰) on whole blood	10.2 ± 0.5	9.7 ± 0.3	13.8 ± 0.8	14.0 ± 0.9
δ^13^C (‰) on whole blood	-17.8 ± 0.6	-18.1 ± 0.4	-19.4 ± 0.7	-20.2 ± 0.9
**Habitat of foraging areas (within FR)**				
Bathymetry (BAT; m)	748.1 ± 394.5	669.6 ± 421.7	612.4 ± 384.3	864.3 ± 457.9
Sea Surface Temperature (SST; °C)	17.5 ± 1.9	18.0 ± 1.5	23.2 ± 1.8	24.8 ± 2.0
Chlorophyll *a* concentration (CHL; mg m^−3^)	1.8 ± 0.7	2.0 ± 0.8	0.7 ± 0.2	0.8 ± 0.3
Wind speed (WSPD; m s^−1^)	6.4 ± 1.8	6.6 ± 1.9	5.1 ± 2.9	4.7 ± 2.8

**Table 2:** Generalized Linear Mixed Effect Models (GLMMs) testing the effect of year (2013 vs 2014) and breeding stage (incubation *vs* chick-rearing) on mean foraging trip characteristics, spatial and trophic ecology parameters of Cape Verde shearwaters *Calonectris edwardsii* shown in Table 1. Forty foraging trips from the chick-rearing period (twenty per year) were randomly selected for statistical purposes (i.e. in order to have similar sample size between breeding phases). The individual was used as a random effect to avoid pseudo-replication issues. Significant results in **bold**.

	Year		Breeding phase	
Variables	GLMM	P	GLMM	P
**Foraging trip characteristics**				
N tracks [N birds]	—	—	—	—
Trip duration (days)	F_1,135_ = 2.46	0.12	F_1,135_ = 16.83	**< 0.001**
Total distance covered (km)	F_1,135_ = 1.49	0.21	F_1,135_ = 19.45	**< 0.001**
Maximum distance from colony (km)	F_1,135_ = 2.19	0.14	F_1,135_ = 20.01	**< 0.001**
Time spent flying trip^−1^ day^−1^(h)	F_1,135_ = 2.75	0.10	F_1,135_ = 11.70	**0.001**
**Spatial ecology parameters**				
Meso-scale Area of Restricted Search (ARS) Radii (km)	F_1,135_ = 0.50	0.48	F_1,135_ = 20.24	**< 0.001**
Meso-scale max. First Passage Time (hours)	F_1,135_ = 2.00	0.16	F_1,135_ = 21.14	**< 0.001**
Distance of meso-scale ARS zone to colony (km)	F_1,135_ = 1.21	0.27	F_1,135_ = 11.81	**0.001**
Coarse-scale Area of Restricted Search (ARS) Radii (km)	F_1,135_ = 0.92	0.34	F_1,135_ = 6.99	**0.01**
Coarse-scale max. First Passage Time (hours)	F_1,135_ = 1.51	0.22	F_1, 135_ = 5.65	**0.02**
Distance of coarse-scale ARS zone to colony (km)	F_1,135_ = 2.59	0.11	F_1,135_ = 17.03	**< 0.001**
FR overlaps within years and within the same breeding stage (%)	F_1,135_ = 0.52	0.47	F_1,135_ = 4.89	**0.03**
FR overlaps among years and within the same breeding stage (%)	—	—	—	—
FR overlaps within breeding stages (%)	—	—	—	—
FR overlaps among breeding stages (%)	—	—	—	—
**Trophic ecology**				
δ^15^N (‰) on whole blood	F_1,41_ = 2.51	0.12	F_1,41_ = 12.57	**0.001**
δ^13^C (‰) on whole blood	F_1,41_ = 5.86	**0.02**	F_1,41_ = 18.58	**< 0.001**
**Habitat of foraging areas (within FR)**				
Bathymetry (BAT; m)	F_1,251_ = 1.12	0.29	F_1,248_ = 1.73	0.19
Sea Surface Temperature (SST; °C)	F_1,251_ = 1.91	0.17	F_1,248_ =	**< 0.001**
Chlorophyll *a* concentration (CHL; mg m^−3^)	F_1,251_ = 1.16	0.29	F_1,248_ =	**< 0.001**
Wind speed (WSPD; m s^−1^)	F_1,251_ = 0.92	0.34	F_1,248_ =	0.21

### Habitat use

All four habitat suitability models showed a good to excellent ability to predict the observed habitat used by Cape Verde shearwaters (all AUC > 0.85; Table 3). Overall, there was a high inter-annual repeatability on the more relevant parameters explaining ARS locations of individuals during the incubation phase. During the chick-rearing phase, there was a higher inter-annual variation on the parameters better explaining the species’ distribution. For both study years, the SST and SSTG were the main triggers of ARS behaviour during incubation, while during chick-rearing the birds foraging distribution was mostly triggered by DCOL (Table 3). All habitat characteristics of foraging regions (50% kernel UD) were similar between years, with birds inhabiting colder (SST) and more productive (CHL) waters during incubation than during chick rearing (Tables 1 and 2). Spatial overlap of the birds’ foraging region (FR; 50% Kernel UD) was always higher during incubation (> 84%) than during chick-rearing (< 77.9%), with the lowest value attained when comparing the FR locations between breeding stages (18%; Table 1).

**Table 3:** Estimates of model fit and relative contributions of the environmental variables to the MaxEnt models generated for the spatial distribution of Cape Verde shearwaters *Calonectris edwardsii* from Raso Islet (Cape Verde) during incubation and chick-rearing of 2013 and 2014. AUC—Area Under the Receiver Operating Curve. Parameters contributing in more than 10% in **bold**.

	Incubation		Chick-rearing	
	2013	2014	2013	2014
Test AUC (%)	91.3	92.8	85.5	89.4
**Parameter contribution (%)**				
Bathymetry (BAT)	**15.9**	**11.5**	—	**11.1**
Sea Surface Temperature (SST)	**36.1**	**34.8**	**10.5**	**10.1**
Chlorophyll *a* concentration (CHL)	—	—	2.4	**19.2**
Gradient in BAT (BATG)	**12.1**	**11.8**	**17.3**	**13.8**
Gradient in SST (SSTG)	**23.1**	**25.7**	6.6	6.1
Gradient in CHL (CHLG)	—	—	**19.2**	—
Wind speed (WSPD)	7.1	—	6.2	—
Distance to colony (DCOL)	4.7	3.9	**40.3**	**27.4**
**Permutation contribution (%)**				
Bathymetry (BAT)	35.5	27.6	—	**25.7**
Sea Surface Temperature (SST)	23.4	26.8	15.9	**8.6**
Chlorophyll *a* concentration (CHL)	—	—	12.7	**10.2**
Gradient in BAT (BATG)	13.5	15.1	5.6	**29.3**
Gradient in SST (SSTG)	14.1	18.4	8.9	**4.7**
Gradient in CHL (CHLG)	—	—	20.8	—
Wind speed (WSPD)	5.9	—	4.1	—
Distance to colony (DCOL)	7.6	12.1	32.0	21.5

### Trophic ecology

During incubation, birds had comparatively narrow isotopic niches (SEAc 2013 = 3.4 and SEAc 2014 = 8.0), with a high inter-annual overlap (SEAc overlap = 96%). When rearing their chick, birds showed the widest breadth of trophic levels (largest range in δ^15^N) and high diversity of basal resources (largest range in δ^13^C), which resulted in wider isotopic niches for both years (SEAc 2013 = 18.1 and SEAc 2014 = 18.9).

Between incubation and chick-rearing, birds significantly increased and decreased respectively their δ^15^N and δ^13^C signatures, with the SEAc size of incubating birds during 2013 being significantly lower than that of chick rearing birds in 2013 (SEA_B_: P = 0.02) and 2014 (SEA_B_: P = 0.02). During 2013 the δ^13^C signature of birds was significantly lower, when compared to 2014 (Tables 1 and 2; Fig 1C).

### Foraging distribution and marine conservation

**Fig 2:**
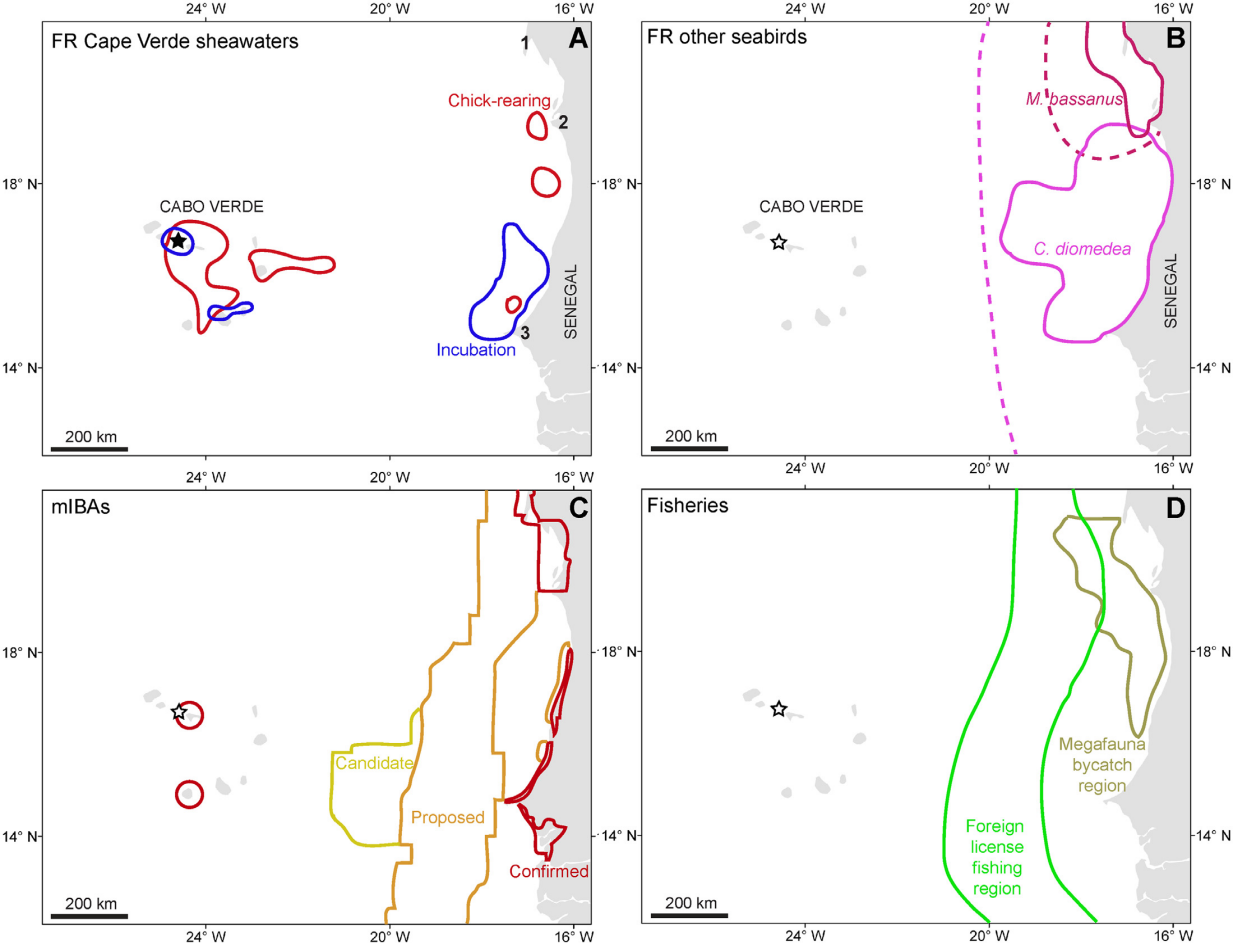
**(A)** Foraging regions (50% kernel UD) of Cape Verde shearwaters *Calonectris edwardsii* during incubation (blue polygons; N = 40 trips from 22 ind.) and chick-rearing (red polygons; N = 97 trips from 21 ind.) periods of 2013 and 2014. 1—Cap Blanc; 2—Southernmost area of the Parc National Du Banc D’Arguin; 3—Cap-Vert, Dakar, Senegal. **(B)** Foraging distribution of Northern gannets *Morus bassanus* (dark pink line; [16]) and Scopoli’s shearwaters *Calonectris diomedea* (light pink line; [33]) tracked with GPS/ PTT-transmitters (continuous line) and GLS devices (dashed line; always from the line limit towards the African coastline). **(C)** Confirmed, proposed and candidate marine Important Bird Areas (mIBAs) (http://maps.birdlife.org/marineIBAs/default.html). **(D)** Identified areas of megafauna bycatch [56] and foreign license fishing region (within lines; [57]).

There was a high overlap of the Cape Verde shearwaters foraging regions (50% kernel UD) with the foraging distribution of related species—Scopoli’s shearwater—during incubation (~70%) and slight overlap during chick-rearing (~7%), while not overlapping at all with the non-related northern gannets during incubation and marginally during chick-rearing (~4%). The overlap with marine IBAs was generally low (max. of 18% for incubating birds; Table 4). During chick-rearing, birds heavily foraged over known areas of megafauna bycatch off West Africa, while avoiding the foreign license fishing region both during incubation and chick-rearing (Table 4; Fig 2).

**Table 4:** Percentage (%) overlap between foraging regions (FR—50% kernel UD) of Cape Verde shearwaters *Calonectris edwardsii* (CVSh) during the breeding period of two study years (2013 and 2014) and (1) foraging distribution of Northern gannets *Morus bassanus* [16] and Scopoli’s shearwaters *Calonectris diomedea* [33] tracked with GPS/ PTT-transmitters; (2) confirmed, proposed and candidate marine Important Bird Areas (mIBAs) (http://maps.birdlife.org/marineIBAs/default.html); (3) identified areas of megafauna bycatch [56] and foreign license fishing region [57], as shown in Fig 2.

**(1) CVSh FR *vs* other seabirds**	**Incubation**	**Chick-rearing**
Northern gannets *M*. *bassanus*	0.0	4.4
Scopoli’s shearwaters *C*. *diomedea*	69.2	6.8
**(2) CVSh FR *vs* marine IBAs**		
Confirmed marine IBAs	18.1	7.6
Proposed marine IBAs	9.3	3.2
Candidate marine IBAs	0.0	0.0
**(3) CVSh FR *vs* fishery activities**		
Areas of megafauna bycatch	2.9	27.3
Foreign licence fishing region	0.0	0.0

## Discussion

Our study provides the first data of the fine-scale foraging distribution, at-sea behaviour and trophic choices of a near endangered seabird species, the Cape Verde shearwater, endemic from the Cape Verde archipelago. Overall, there was an apparent inter-annual consistency on the spatial, foraging and trophic ecology and an obvious alteration on the foraging strategies of adult breeders among breeding phases (i.e. from incubation to chick-rearing). Though only future data collection (comprising at least 3–4 years of data) will attest if this consistency is maintained through time. During incubation, birds mostly targeted a discrete region off West Africa (in front of Dakar, Senegal), known by their enhanced productivity profile and thus also highly used by other marine predators, notably migratory seabirds from Europe (e.g. [15]), and heavily exploited by international industrial fishery fleets. When provisioning their chick, adults exploited the comparatively less productive tropical environment, at relatively close distance from their breeding colony. Plus, birds enlarged their trophic niche and increased the trophic level of their prey from incubation to chick-rearing. Moreover, the species exhibited a clear dual foraging strategy, performing mostly short (≤ 3 days duration) foraging excursions to provision their growing chick and few long (> 3 days duration) excursions to replenish their own reserves and body condition [58,59].

### Oceanographic cues triggering the ARS behaviour

When studying the foraging ecology of top predators, species distribution models are an efficient tool to link behavioural decisions with oceanographic scale-dependent processes [2]. From our habitat models, sea floor depth (both DEP and DEPG) and SST were the variables that kept explaining variation in FPT over the different habitats exploited by the species. This species relies mostly on small pelagic fish and cephalopods [21], which are usually more abundant in neritic (shallow water) than in oceanic (deep water) environments. Besides, SST seems to be the environmental proxy of productivity most used in previous studies, triggering the foraging behaviour of marine top predators in a diversity of marine systems [60].

During the incubation period, tracked individuals regularly commuted to off West Africa, with long trips comprising ~ 75% of the overall trips during this period, to forage extensively over this very productive region [54]. Such straight commuting movement reveals that birds have learnt where there are consistent resources and is believed to be the most efficient movement to search for prey over large scales [61]. In fact, oceanographic phenomena triggering the ARS behaviour of individuals foraging off West Africa, such as steep bathymetric areas (BATG) or frontal regimes (CHLG and SSTG) usually occur at a large spatio-temporal scale (i.e. hundred km and for several days) [62]. Birds coped with this by displaying maximum FPT of ~ 38h, at about 73km (ARS radii). At this scale (10s km) enhancement of ocean productivity and concentration of prey and predators is supposed to be maintained by hydrographical (e.g. fronts) and physical (e.g. seamount slopes) features [63]. Moreover, mean values of meso-scale FPT and ARS areas radii closely resembled those of the related Cory’s shearwaters breeding at Selvagem Grande, and exploiting the Canary Current (CC) system further north, off Mauritania [2]. Short-tailed albatrosses *Phoebastria albatrus* breeding at Torishima and foraging over both oceanic and neritic domains of the Pacific ocean [64] displayed similar spatio-temporal scales of ARS. Besides, the extra effort of commuting to a distant environment (e.g. higher total distance covered and time spent flying) should be rewarded by increased availability of ‘natural prey’ items over such region. Plus, the availability of extra food subsidies (besides ‘natural prey’) provided by fishery discards, might be another motive to embark repeatedly in such (comparatively) long journey, as we know that closely related *Calonectris* species also rely intermittently on fishery subsidies [65].

During chick-rearing, seabird breeders are generally constrained to find food resources at short distance from their colony, in order to regularly visit the nest and successfully raise their chick. Tropical waters, such as those surrounding the Cape Verde archipelago, are characterized by low productivity profiles [1]. Cape Verde shearwaters seem to overcome this issue by optimizing foraging strategies and targeting (1) the inter-islands channels, were productivity should be enhanced by the more intense and nutrient-enriched currents (higher CHLG; [66]) and, (2) some specific seamounts (low BAT) at short distance from their breeding location, where an enhancement in productivity should occur, through a local upwelling phenomena [67]. Despite this more frequent pattern (~77% short trips in the colony surroundings), birds also performed few long trips (the remaining ~23% of trips) to exploit productive (high CHL, low SST) and shallow (low BAT) regions and areas with greater slope (higher BATG) off West Africa (similar to the incubation pattern, but further north within the CC system). Still during chick-rearing, adults significantly shifted their foraging pattern (when compared to incubation) most likely responding to the urge to feed their chick. Such shift in behaviour included lower values of almost all foraging parameters. This is in line with the foraging strategies of other pelagic seabird species during this phase (e.g. [30]). Mean values of coarse-scale FPT and ARS were similar to those of Cory’s shearwaters breeding at Berlenga [2] and northern gannets *Morus bassanus* breeding at Bass Rock [68], which foraged also (mostly) within their colony surroundings.

### Isotopic niches of incubating and chick-rearing birds

In the marine environment, the distribution of nitrogen and carbon isotopes varies geographically [69], which directly shapes the trophic niche of prey inhabiting a specific location [70] and predators feeding on those prey [48]. δ^13^C values usually separates consumers feeding habits in coastal and benthic environments (more enriched) from oceanic and pelagic habitats (more depleted; [48]). The exploitation of marine resources at more coastal areas off West Africa, most likely shaped the lower carbon isotopic signature of individuals during incubation, thus isotopically segregating such group from birds during chick-rearing. Besides, when foraging off West Africa, chick-rearing birds foraged at higher latitudes when compared to incubating birds, (during both years). This might have lowered δ^13^C values, because carbon isotopic signatures at the base of the marine food-web are supposed to decrease with increasing latitude [48]. Furthermore, a possible higher consumption of demersal prey-fish discarded by fishing vessels, to which Cape Verde shearwaters are known to attend in very large numbers [18], may have also lowered δ^13^C values. Though demersal species were not detected on the species’ diet composition in 2012 and 2013 [21]. Plus, the large numbers of birds reported to attend fishery discards [18], might be mainly composed by non-breeders (juveniles, sabbaticals and failed breeders) instead of active breeders. Non-breeders usually represent an important part of seabird populations, and their attendance to fishing vessels represent and extra motive of concern for the species conservation, through a potential increase in the numbers of by-caught individuals and consequent decrease in the recruitment rate of younger individuals into the breeding population. Interestingly, during incubation birds showed a narrow and highly overlapping isotopic niche among study years, while chick-rearing birds enlarged their isotopic niche, increased the trophic level (i.e. higher δ^15^N) and showed low overlap between years. This niche enlargement and (possible) diversification of the origin and species of prey, might be a response to the nutritional requirements of their growing chick [31].

### Conservation considerations

Cape Verde shearwaters face well identified threats: (1) on-land, the species has been harvested for food and bait for a long time (probably several centuries) especially at its main breeding aggregations (i.e. Raso and Branco Islets); at-sea, the species survival is currently at jeopardy from (2) unintentional (by-catch) and (3) intentional (illegal harvest) killings in fishing gears, off West Africa, within Cape Verde national waters and at their main wintering site (off Brazil; [26]); still at-sea, the species may (4) face high competition for resources (to access available pelagic prey species) and (5) feed on fisheries discards and offal. Being a long-distance migratory species, Cape Verde shearwaters face threats such as by-catch [26] over a large geographical range. Though there is a limited knowledge about to what extent those threats impact the population, they are certainly key determinants in the overall population dynamics. The urge to gather information about the potential impact of fisheries on marine wildlife off West Africa has been raised by several NGO’s (e.g. ‘MAVA—Fondation pour la nature’) and seabird researchers (e.g. [56]). In this respect, the recent report of eight illegal containers with (potentially) tens of thousands of frozen seabirds, boxed and labelled as fish and ready to ship to Asia (Kees Camphuysen, *pers*. *com*. *in* [16]) is a serious matter. This should concern not only conservationists and researchers, but also state authorities with jurisdiction on the area, which should control harvesting activities of target and non-target marine wildlife [16]. This might be achieved by (1) clarifying the legal status of fisheries operating within the CC system (i.e. both off West Africa and within the Cape Verde EEZ), (2) designating marine protected areas (MPAs), fostered by the marine Important Bird Areas (IBAs) already identified for the region (http://maps.birdlife.org/marineIBAs/default.html) and the increasing amount of tracking data from marine predators, to refine limits of those areas of conservation concern (e.g. this study), (3) Taking into account scientific projects gathering knowledge on the strategic combination of fisheries and ecosystem governance frameworks, such as the Canary Current Large Marine Ecosystem project (CCLME; http://www.canarycurrent.org/en) and (4) investing on marine surveillance means within those designated MPAs. Such management actions will just be fully effective if along with them there is a change in mentality from the European authorities, to stop through legislation, the current frantic rush to harvest fish stocks off the West African coast [6]. In fact, the former confiscated shipment might represent ‘the tip of an iceberg’ of wider illegal fishery actions threatening West African marine economies and food security [71,72].

Our study shows that the Cape Verde shearwater is a suitable sentinel species of the marine ecosystem health and might be a useful umbrella species, for the conservation of other aerial and aquatic marine taxa inhabiting off West Africa and within Cape Verde national waters. This is because the species (1) as a broad at-sea distribution within the area, thus targeting diverse oceanographic features (this study), natural enhancers of productivity and also targeted by other marine taxa [56], (2) feeds on the most abundant commercial fish species, such as sardinella *Sardinella* sp or bigeye scad *Selar crumenophthalmus* species [21,22], hence functioning as a bio-indicator of possible changes on the marine trophic webs [73] and, (3) overlaps in distribution (just spatially, not temporally) with other seabird taxa, belonging to different ecological guilds. Namely, northern gannets [16], Scopoli’s shearwaters [33], Cory’s shearwaters [25,74,75], Macaronesian shearwaters *Puffinus baroli* [76], Deserta’s petrels *Pterodroma deserta* [77], Zino’s petrels *Pterodroma madeira* [78], Sabine’s Gulls *Larus sabini* [79] and long-tailed Skuas *Stercorarius longicaudus* [80]. Nevertheless, it’s effectiveness as umbrella species for marine conservation should only be proven with further data collection in the coming years. Such collection of ecological information should not be restricted to tracking data and bird tissues for SIA, but also diet samples along the breeding period to better investigate the species feeding ecology and it’s possible consumption of fisheries discards. The small overlap between the foraging regions of Cape Verde shearwaters off West Africa and the confirmed, proposed and candidate marine Important Bird Areas (IBAs) indicates that much work is still needed in identifying marine IBAs in this region. We envisage that the new knowledge provided by this work is valuable to better delineate such areas and the ‘fisheries-conservation hotspots’ at a regional scale [4].
